# Crystal structure of a polymorph of *μ*-oxido-bis­[(5,10,15,20-tetra­phenyl­porphyrinato)iron(III)]

**DOI:** 10.1107/S2056989019007576

**Published:** 2019-05-31

**Authors:** Morten K. Peters, Christian Näther, Rainer Herges

**Affiliations:** a Otto-Diels-Institut für Organische Chemie, Christian-Albrechts-Universität Kiel, Otto-Hahn-Platz 4, D-24098 Kiel, Germany; b Institut für Anorganische Chemie, Christian-Albrechts-Universität Kiel, Max-Eyth Str. 2, D-24118 Kiel, Germany

**Keywords:** crystal structure, ferric porphyrin, dimer, polymorphism, twinning

## Abstract

The title compound, [Fe_2_(C_44_H_28_N_4_O)_2_O], was obtained as a by-product during the synthesis of Fe^III^ tetra­phenyl­porphyrin perchlorate. It crystallizes as a new polymorphic modification in addition to the ortho­rhom­bic form previously reported [Hoffman *et al.* (1972 ▸). *J. Am. Chem. Soc.* **94**, 3620–3626; Swepston & Ibers (1985 ▸) *Acta Cryst.* C**41**, 671–673; Kooijmann *et al.* (2007 ▸). Private Communication (refcode 667666). CCDC, Cambridge, England]. In its crystal structure, the two crystallographically independent Fe^III^ cations are coordinated in a square-planar environment by the four N atoms of a tetra­phenyl­porphyrin ligand. The Fe^III^-tetra­phenyl­porphyrine units are linked by a μ_2_-oxido ligand into a dimer with an Fe—O—Fe angle close to linearity. The final coordination sphere for each Fe^III^ atom is square-pyramidal with the μ_2_-oxido ligand in the apical position. The crystal under investigation consisted of two domains in a ratio of 0.691 (3): 0.309 (3).

## Abstract 2

In the crystal structure of the new polymorph of of μ-oxido-bis­[(5,10,15,20-tetra­phenyl­porphyrinato)iron(III)], two Fe^III^ tetra­phenyl­porphyrin units are linked by μ_2_-oxido O atoms into dimers, leading to a square-pyramidal coordination for each of the cations.

## Chemical context   

Porphyrins have a wide range of applications. For example, they are useful in photodynamic therapy (PDT) (Ethirajan *et al.*, 2011 ▸; Bonnett, 1995 ▸; Peters *et al.*, 2018*a* ▸), as powerful catalysts in reduction processes in nature and in technologically important reactions (Li & Zamble, 2009 ▸; Peters & Herges, 2018 ▸; Gosden *et al.*, 1978 ▸), or as responsive contrast agents in functional magnetic resonance imaging (*f*MRI) (Venkataramani *et al.*, 2011 ▸; Dommaschk *et al.*, 2015 ▸; Peters *et al.*, 2018*b* ▸).

In a previous publication, we have reported the first light-controlled mol­ecular spin switch based on Fe^III^ tetra­phenyl­porphyrin perchlorate (FeTPPClO_4_) (Shankar *et al.*, 2018 ▸). The starting material FeTPPClO_4_ exists in the admixed-spin state (*S* = 3/2, 5/2). However, in a solution of acetone/dimethyl sulfoxide, a high-spin (*S* = 5/2) complex is formed (Shankar *et al.*, 2018 ▸). The low-spin (*S* = 1/2) state can be induced by a photoswitchable azo­pyridine ligand and can be reversibly switched to the high-spin state by exposure to light (Shankar *et al.*, 2018 ▸; Peters *et al.*, 2019 ▸). This system is reversible by using dimethyl sulfoxide and is neither oxygen nor water sensitive, and no fatigue was observed after more than 1000 switching cycles (Shankar *et al.*, 2018 ▸). Unfortunately, without dimethyl sulfoxide, the switching is not reversible and a by-product is formed as indicated from the shift of the pyrrol protons observed in an NMR experiment. The amount of this by-product increases with increasing reaction time. To identify the nature of this by-product, we tried to obtain single crystals after very long reaction times, but without any success. If, however, 4-methyl­imidazole is used instead of a azo­pyridine ligand, dark red-coloured crystals of the same by-product were obtained. The crystals were subjected to single-crystal X-ray diffraction analysis, revealing that a dimer has formed where two Fe^III^ cations are bridged by a *μ*_2_-oxido ligand. The source of oxygen is still unknown but it is likely that it possibly originates from water or from hygroscopic 4-methyl­imidazole. It is noted that a crystal structure of this compound has already been reported (Strauss *et al.*, 1987 ▸) but this form crystallizes in the ortho­rhom­bic space group *Aba*2 (Hoffman *et al.*, 1972 ▸; Swepston & Ibers, 1985 ▸; Kooijmann *et al.* 2007 ▸). Therefore, the new polymorph of the title compound was further investigated, and its crystal structure is reported in this communication.
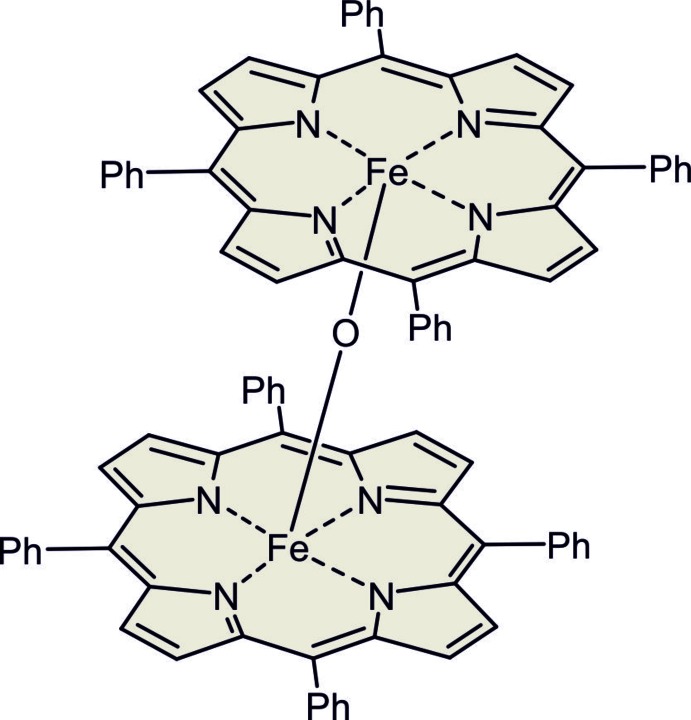


## Structural commentary   

**Figure 1:**
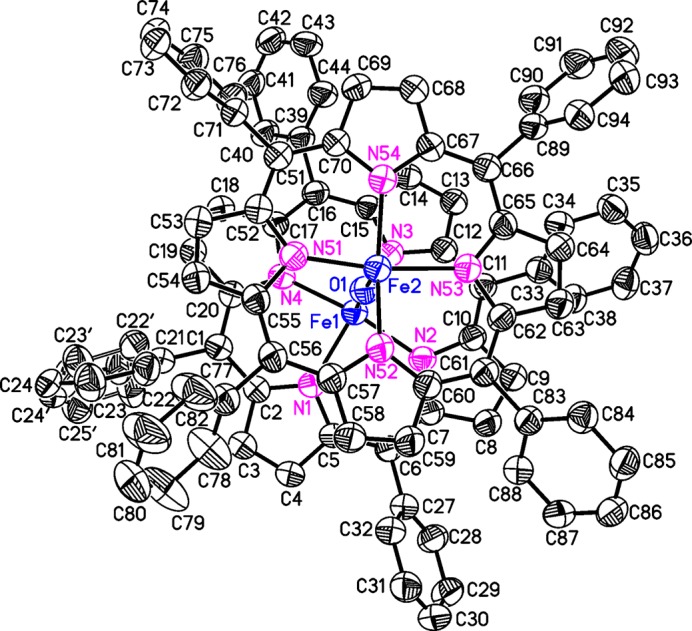
Mol­ecular structure of the title compound with atom labelling and displacement ellipsoids drawn at the 50% probability level. The H atoms are omitted for clarity; the disorder of one of the phenyl rings is shown with full and open bonds.

**Figure 2:**
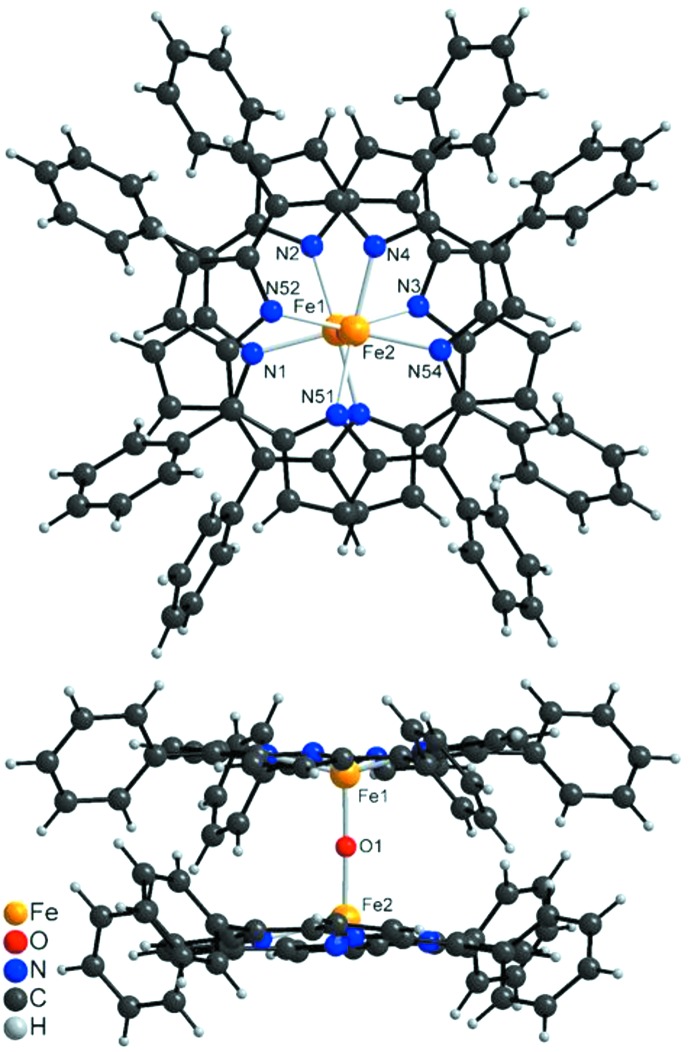
Top and side view of the mol­ecular structure of the title compound showing the coordination around the Fe^III^ atoms. The disorder of one of the phenyl rings is omitted for clarity.

In the crystal structure of the triclinic polymorph of the title compound, the two crystallographically independent Fe^III^ cations are each coordinated by the four N atoms of tetra­phenyl­porphyrin ligands in a square-planar environment (Figs. 1 ▸ and 2 ▸). These complexes are linked into dimers *via* a *μ*_2_-oxido O atom, leading to a final square-pyramidal coordination for each of the Fe^III^ cations (Fig. 2 ▸), with τ_5_ values (Addison *et al.*, 1984 ▸) of 0.04 (Fe1) and 0.01 (Fe2), indicating only slight deviations from the ideal geometry for which τ_5_ = 0. For Fe1 the Fe—N bond lengths are very similar, whereas for Fe2 they are slightly different (Table 1 ▸). There are also small differences in the Fe—O distances, which shows that the bridge is not symmetrical [the Fe—O—Fe angle is 177.71 (18)°]. This is in contrast to the ortho­rhom­bic form where both Fe—O distances are identical because of symmetry restrictions as this complex is located on a twofold rotation axis (Hoffman *et al.*, 1972 ▸; Swepston & Ibers, 1985 ▸; Kooijmann *et al.* 2007 ▸). Nevertheless, the ortho­rhom­bic form likewise shows a small distortion of the coordination polyhedron around Fe^III^, and in both modifications the Fe^III^ cations are shifted out of the porphyrine plane in direction towards the O atoms [0.366 (1) Å for Fe1 and 0.399 (1) Å for Fe2 in the monoclinic structure of the title compound; Fig. 2 ▸]. The porphyrine ring planes in the title compound are rotated by 28.5 (5)° against each other, whereas in the ortho­rhom­bic form they exhibit an almost staggered arrangement of the Fe—N bonds, close to *D*_4*d*_ symmetry.

## Supra­molecular features   

**Figure 3:**
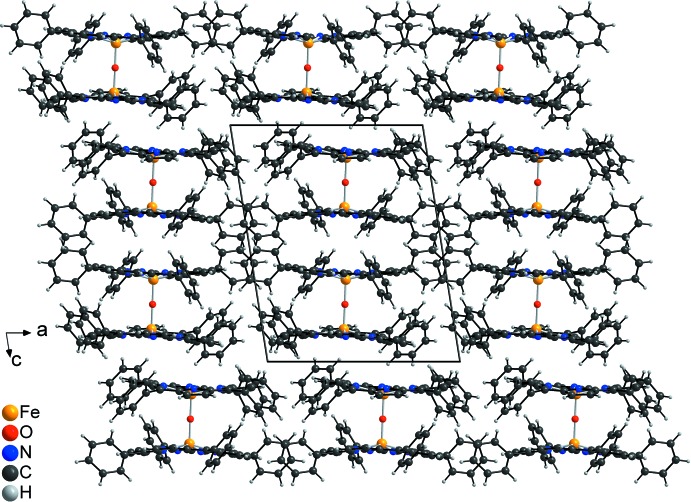
Crystal structure of the title compound in a view along the *b* axis. The disorder of one of the phenyl rings is omitted for clarity.

In the crystal structure of the title compound, the dimers are arranged in columns that elongate parallel to the *b* axis (Fig. 3 ▸). There are no hydrogen bonds between the dimers, and there is also no hint of significant π–π inter­actions. Therefore, the packing appears to be dominated by non-directed van der Waals inter­actions. It is noted that the packing of the dimers is completely different in the two polymorphic forms. In the ortho­rhom­bic form, the dimers are also arranged in columns but neighbouring columns are shifted relative to each other; for comparison of the two polymorphs, see Figs. 3 ▸ and 4 ▸. The density of the triclinic polymorph is slightly higher than that of the ortho­rhom­bic form, indicating that the former most probably represents the thermodynamic stable form at absolute zero.

## Database survey   

According to a search in the Cambridge Structural Database (CSD, version 5.40, updated Feb. 2019; Groom *et al.*, 2016 ▸), 1010 structures with iron porphyrins have been reported. Similar *μ*_2_-oxido-bridged iron porphyrins are known. For example, (*μ*_2_-oxido)-bis­(5,10,15,20-tetra­phenyl­porph­yr­in­ato)iron(III) with C_70_ fullerene (Konarev *et al.*, 2010 ▸) and C_60_ fullerene (Litvinov *et al.*, 2003 ▸, 2004 ▸). Other *μ*_2_-oxido iron porphyrins include 5,10,15,20-tetra-*p*-tolyl­porphyrinato)iron(III) (Li *et al.*, 1999 ▸), 5,10,15,20-tetra­kis­(penta­fluoro­phen­yl)porphinatoiron(III) (Gold *et al.*, 1988 ▸), tetra­kis­(2,6-di­fluoro­phen­yl)porphyrinato)iron(III) (Karlin *et al.*, 1994 ▸), 5,10,15,20-tetra­kis­(4-bromo­phen­yl)porphyrinato)iron(III) (Hou *et al.*, 2015 ▸) and 5,10,15,20-tetra­kis­(4-chloro­phen­yl)porphyrinato)iron(III) (Jiao *et al.*, 1997 ▸). As already noted, an ortho­rhom­bic polymorph of the title compound has previously been structurally characterized (Hoffman *et al.*, 1972 ▸; Swepston & Ibers, 1985 ▸; Kooijmann *et al.*, 2007 ▸).

## Synthesis and crystallization   

FeTPPClO_4_ was synthesized as reported (Shankar *et al.*, 2018 ▸). The layering technique was used for crystallization. The lower layer consisted of FeTPPClO_4_ dissolved in di­chloro­methane to which 50 µl 4-methyl­imidazole were added, and *n*-heptane was used as the upper anti­solvent.

## Refinement   

Crystal data, data collection and structure refinement details are summarized in Table 2 ▸.

All crystals consisted of more than one domain, but the structure could be solved in space group *P* 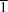 neglecting the presence of two domains. However, these refinement runs led to poor reliability factors and several electron density maxima were observed that could not be resolved. The TwinRotMat option in *PLATON* (Spek, 2009 ▸) suggested a twofold rotation axis as twin element with the matrix (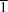 0 0, 0 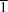 0, −0.389, −0.663 1). Several data sets in HKLF-5 format were generated using different sizes of the integration box in *X-AREA* (Stoe, 2008 ▸) and different overlap criteria in *PLATON* (Spek, 2009 ▸) until the best data set was obtained. The final refinement using this data set led to a ratio of the two domains of 0.691 (3): 0.309 (3) and acceptable reliability factors.

The C—H hydrogen atoms were located in a difference Fourier map but were positioned with idealized geometry and refined with with *U*_iso_(H) = 1.2*U*_eq_(C) using a riding model with C—H = 0.95 Å. One of the phenyl rings is disordered over two orientations (ratio 0.55:0.45) and was refined using a split model with restraints for the bond lengths (DFIX).

## Supplementary Material

Crystal structure: contains datablock(s) I. DOI: 10.1107/S2056989019007576/wm5503sup1.cif

Structure factors: contains datablock(s) I. DOI: 10.1107/S2056989019007576/wm5503Isup2.hkl

CCDC reference: 1918323

Additional supporting information:  crystallographic information; 3D view; checkCIF report

## Figures and Tables

**Figure 4:**
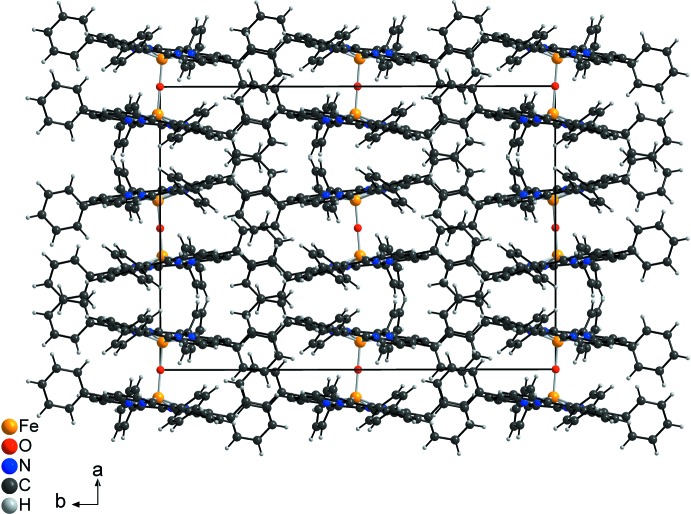
Crystal structure of the ortho­rhom­bic form of the title compound in a view along the *c* axis.

**Table 1:** Selected geometric parameters (Å, °)

Fe1—O1	1.766 (3)	O1—Fe2	1.757 (3)
Fe1—N3	2.069 (3)	Fe2—N51	2.078 (3)
Fe1—N2	2.078 (3)	Fe2—N53	2.080 (3)
Fe1—N1	2.079 (3)	Fe2—N54	2.084 (3)
Fe1—N4	2.084 (3)	Fe2—N52	2.091 (3)
			
O1—Fe1—N3	103.31 (13)	O1—Fe2—N51	103.46 (13)
O1—Fe1—N2	102.11 (13)	O1—Fe2—N53	104.50 (13)
N3—Fe1—N2	87.38 (13)	N51—Fe2—N53	152.04 (13)
O1—Fe1—N1	103.37 (13)	O1—Fe2—N54	103.78 (13)
N2—Fe1—N1	87.14 (13)	N51—Fe2—N54	86.63 (13)
O1—Fe1—N4	102.12 (13)	N53—Fe2—N54	86.85 (13)
N3—Fe1—N4	87.27 (13)	O1—Fe2—N52	103.63 (13)
N2—Fe1—N4	155.77 (13)	N51—Fe2—N52	86.99 (13)
N1—Fe1—N4	87.10 (13)	N53—Fe2—N52	86.40 (13)
Fe2—O1—Fe1	177.71 (18)	N54—Fe2—N52	152.59 (13)

**Table 2:** Experimental details

Crystal data	
Chemical formula	[Fe_2_(C_44_H_28_N_4_O)_2_O]
*M*_r_	1353.10
Crystal system, space group	Triclinic, *P* 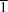
Temperature (K)	170
*a*, *b*, *c* (Å)	14.4477 (4), 14.5325 (4), 17.9076 (5)
α, β, γ (°)	71.266 (2), 75.725 (2), 70.506 (2)
*V* (Å^3^)	3315.42 (17)
*Z*	2
Radiation type	Mo *K*α
μ (mm^−1^)	0.50
Crystal size (mm)	0.3 × 0.2 × 0.15

Data collection	
Diffractometer	Stoe IPDS2
No. of measured, independent and observed [*I* > 2σ(*I*)] reflections	14436, 14436, 12017
(sin θ/λ)_max_ (Å^−1^)	0.639

Refinement	
*R*[*F* ^2^ > 2σ(*F* ^2^)], *wR*(*F* ^2^), *S*	0.075, 0.214, 1.05
No. of reflections	14436
No. of parameters	938
No. of restraints	12
H-atom treatment	H-atom parameters constrained
Δρ_max_, Δρ_min_ (e Å^−3^)	0.52, −0.67

Computer programs: *X-AREA* (Stoe, 2008 ▸), *SHELXT* (Sheldrick, 2015*a* ▸), *SHELXL2014* (Sheldrick, 2015*b* ▸), *XP* in *SHELXTL* (Sheldrick, 2008 ▸), *DIAMOND* (Brandenburg, 2014 ▸) and *publCIF* (Westrip, 2010 ▸).

## supplementary crystallographic information

### Crystal data

**Table:**

[Fe_2_(C_44_H_28_N_4_)O]	*Z* = 2
*M**_r_* = 1353.10	*F*(000) = 1400
Triclinic, *P*1	*D*_x_ = 1.355 Mg m^−^^3^
*a* = 14.4477 (4) Å	Mo *K*α radiation, λ = 0.71073 Å
*b* = 14.5325 (4) Å	Cell parameters from 37885 reflections
*c* = 17.9076 (5) Å	θ = 1.2–27.0°
α = 71.266 (2)°	µ = 0.50 mm^−^^1^
β = 75.725 (2)°	*T* = 170 K
γ = 70.506 (2)°	Block, dark red
*V* = 3315.42 (17) Å^3^	0.3 × 0.2 × 0.15 mm

### Data collection

**Table:**

Stoe IPDS-2 diffractometer	θ_max_ = 27.0°, θ_min_ = 1.2°
ω scans	*h* = −18→18
14436 measured reflections	*k* = −18→18
14436 independent reflections	*l* = −15→22
12017 reflections with *I* > 2σ(*I*)	

### Refinement

**Table:**

Refinement on *F*^2^	12 restraints
Least-squares matrix: full	Hydrogen site location: inferred from neighbouring sites
*R*[*F*^2^ > 2σ(*F*^2^)] = 0.075	H-atom parameters constrained
*wR*(*F*^2^) = 0.214	*w* = 1/[σ^2^(*F*_o_^2^) + (0.0892*P*)^2^ + 3.7143*P*] where *P* = (*F*_o_^2^ + 2*F*_c_^2^)/3
*S* = 1.05	(Δ/σ)_max_ < 0.001
14436 reflections	Δρ_max_ = 0.52 e Å^−^^3^
938 parameters	Δρ_min_ = −0.67 e Å^−^^3^

### Special details

**Table:**

Geometry. All esds (except the esd in the dihedral angle between two l.s. planes) are estimated using the full covariance matrix. The cell esds are taken into account individually in the estimation of esds in distances, angles and torsion angles; correlations between esds in cell parameters are only used when they are defined by crystal symmetry. An approximate (isotropic) treatment of cell esds is used for estimating esds involving l.s. planes.
Refinement. Refined as a two-component twin

### Fractional atomic coordinates and isotropic or equivalent isotropic displacement parameters (Å^2^)

**Table:**

	*x*	*y*	*z*	*U*_iso_*/*U*_eq_	Occ. (<1)
Fe1	0.57714 (4)	0.35357 (4)	0.13707 (3)	0.04270 (15)	
O1	0.5511 (2)	0.3176 (2)	0.24234 (16)	0.0487 (6)	
N1	0.7312 (2)	0.3020 (3)	0.1133 (2)	0.0475 (7)	
N2	0.5944 (2)	0.4969 (2)	0.1144 (2)	0.0457 (7)	
N3	0.4378 (2)	0.4242 (2)	0.1037 (2)	0.0462 (7)	
N4	0.5732 (2)	0.2275 (3)	0.1077 (2)	0.0451 (7)	
C1	0.7471 (3)	0.1258 (3)	0.1179 (3)	0.0514 (9)	
C2	0.7848 (3)	0.2041 (3)	0.1138 (3)	0.0500 (9)	
C3	0.8882 (3)	0.1932 (4)	0.1092 (3)	0.0564 (10)	
H3	0.9412	0.1335	0.1074	0.068*	
C4	0.8964 (3)	0.2839 (3)	0.1079 (3)	0.0530 (10)	
H4	0.9561	0.2999	0.1047	0.064*	
C5	0.7978 (3)	0.3512 (3)	0.1123 (2)	0.0468 (8)	
C6	0.7743 (3)	0.4499 (3)	0.1189 (2)	0.0467 (8)	
C7	0.6788 (3)	0.5167 (3)	0.1221 (2)	0.0476 (9)	
C8	0.6514 (3)	0.6133 (3)	0.1381 (3)	0.0511 (9)	
H8	0.6953	0.6448	0.1456	0.061*	
C9	0.5522 (3)	0.6520 (3)	0.1408 (3)	0.0524 (9)	
H9	0.5131	0.7146	0.1517	0.063*	
C10	0.5168 (3)	0.5798 (3)	0.1240 (2)	0.0469 (8)	
C11	0.4176 (3)	0.5938 (3)	0.1179 (2)	0.0471 (8)	
C12	0.3838 (3)	0.5233 (3)	0.1026 (2)	0.0468 (8)	
C13	0.2858 (3)	0.5409 (3)	0.0863 (3)	0.0501 (9)	
H13	0.2346	0.6029	0.0812	0.060*	
C14	0.2801 (3)	0.4530 (3)	0.0796 (3)	0.0499 (9)	
H14	0.2243	0.4420	0.0684	0.060*	
C15	0.3739 (3)	0.3796 (3)	0.0924 (2)	0.0457 (8)	
C16	0.3958 (3)	0.2770 (3)	0.0950 (2)	0.0472 (8)	
C17	0.4880 (3)	0.2063 (3)	0.1043 (2)	0.0457 (8)	
C18	0.5099 (3)	0.1001 (3)	0.1115 (3)	0.0531 (10)	
H18	0.4645	0.0655	0.1116	0.064*	
C19	0.6082 (3)	0.0583 (3)	0.1181 (3)	0.0539 (10)	
H19	0.6441	−0.0108	0.1234	0.065*	
C20	0.6472 (3)	0.1382 (3)	0.1157 (2)	0.0474 (9)	
C21	0.8180 (3)	0.0232 (3)	0.1272 (3)	0.0550 (10)	
C22	0.8500 (9)	−0.0263 (8)	0.2022 (7)	0.062 (3)	0.55
H22	0.8255	0.0073	0.2437	0.074*	0.55
C23	0.9166 (11)	−0.1232 (11)	0.2173 (11)	0.072 (4)	0.55
H23	0.9402	−0.1528	0.2671	0.086*	0.55
C24	0.9477 (14)	−0.1756 (13)	0.1589 (9)	0.056 (4)	0.55
H24	0.9860	−0.2444	0.1697	0.067*	0.55
C25	0.9211 (10)	−0.1244 (8)	0.0851 (8)	0.070 (3)	0.55
H25	0.9487	−0.1560	0.0426	0.084*	0.55
C26	0.8556 (11)	−0.0283 (9)	0.0701 (9)	0.070 (4)	0.55
H26	0.8365	0.0023	0.0187	0.084*	0.55
C22'	0.8218 (11)	−0.0500 (10)	0.1954 (10)	0.083 (6)	0.45
H22'	0.7743	−0.0383	0.2410	0.100*	0.45
C23'	0.8935 (13)	−0.1424 (13)	0.2014 (14)	0.086 (6)	0.45
H23'	0.8945	−0.1940	0.2501	0.103*	0.45
C24'	0.9640 (16)	−0.1580 (15)	0.1345 (12)	0.057 (5)	0.44
H24'	1.0189	−0.2169	0.1403	0.068*	0.44
C25'	0.9566 (12)	−0.0911 (11)	0.0608 (11)	0.083 (5)	0.45
H25'	0.9997	−0.1071	0.0145	0.099*	0.45
C26'	0.8833 (11)	0.0018 (12)	0.0560 (10)	0.072 (5)	0.45
H26'	0.8767	0.0504	0.0060	0.086*	0.45
C27	0.8573 (3)	0.4845 (3)	0.1267 (2)	0.0477 (9)	
C28	0.8822 (3)	0.5697 (4)	0.0720 (3)	0.0572 (10)	
H28	0.8467	0.6063	0.0285	0.069*	
C29	0.9591 (4)	0.6004 (4)	0.0815 (3)	0.0664 (12)	
H29	0.9755	0.6583	0.0443	0.080*	
C30	1.0118 (4)	0.5479 (4)	0.1443 (3)	0.0661 (12)	
H30	1.0642	0.5696	0.1502	0.079*	
C31	0.9882 (3)	0.4648 (4)	0.1977 (3)	0.0623 (11)	
H31	1.0245	0.4286	0.2408	0.075*	
C32	0.9117 (3)	0.4325 (3)	0.1899 (3)	0.0535 (10)	
H32	0.8961	0.3746	0.2278	0.064*	
C33	0.3424 (3)	0.6881 (3)	0.1312 (2)	0.0474 (8)	
C34	0.2621 (3)	0.6829 (4)	0.1923 (3)	0.0542 (10)	
H34	0.2551	0.6186	0.2241	0.065*	
C35	0.1920 (3)	0.7693 (4)	0.2082 (3)	0.0624 (11)	
H35	0.1380	0.7638	0.2506	0.075*	
C36	0.2010 (4)	0.8631 (4)	0.1621 (3)	0.0630 (12)	
H36	0.1523	0.9224	0.1721	0.076*	
C37	0.2809 (4)	0.8711 (4)	0.1012 (3)	0.0616 (11)	
H37	0.2877	0.9358	0.0702	0.074*	
C38	0.3512 (3)	0.7838 (3)	0.0856 (3)	0.0523 (9)	
H38	0.4056	0.7895	0.0436	0.063*	
C39	0.3128 (3)	0.2391 (3)	0.0918 (3)	0.0518 (9)	
C40	0.3189 (3)	0.1953 (3)	0.0312 (3)	0.0556 (10)	
H40	0.3754	0.1914	−0.0095	0.067*	
C41	0.2431 (4)	0.1574 (4)	0.0301 (4)	0.0671 (13)	
H41	0.2475	0.1283	−0.0117	0.080*	
C42	0.1617 (4)	0.1616 (4)	0.0890 (4)	0.0697 (14)	
H42	0.1103	0.1350	0.0882	0.084*	
C43	0.1544 (3)	0.2043 (4)	0.1492 (4)	0.0663 (13)	
H43	0.0981	0.2066	0.1900	0.080*	
C44	0.2295 (3)	0.2445 (3)	0.1507 (3)	0.0576 (11)	
H44	0.2234	0.2752	0.1918	0.069*	
Fe2	0.52632 (4)	0.28620 (4)	0.34713 (3)	0.04271 (15)	
N51	0.5793 (2)	0.1295 (2)	0.3793 (2)	0.0457 (7)	
N52	0.6548 (2)	0.2886 (2)	0.3796 (2)	0.0462 (7)	
N53	0.4596 (2)	0.4245 (2)	0.3749 (2)	0.0447 (7)	
N54	0.3845 (2)	0.2660 (2)	0.3737 (2)	0.0463 (7)	
C51	0.4304 (3)	0.0883 (3)	0.3684 (2)	0.0480 (9)	
C52	0.5318 (3)	0.0641 (3)	0.3736 (3)	0.0478 (9)	
C53	0.6006 (3)	−0.0334 (3)	0.3762 (3)	0.0553 (10)	
H53	0.5866	−0.0913	0.3729	0.066*	
C54	0.6895 (3)	−0.0286 (3)	0.3842 (3)	0.0572 (10)	
H54	0.7490	−0.0825	0.3888	0.069*	
C55	0.6769 (3)	0.0734 (3)	0.3846 (2)	0.0476 (9)	
C56	0.7525 (3)	0.1104 (3)	0.3891 (3)	0.0483 (9)	
C57	0.7402 (3)	0.2102 (3)	0.3889 (3)	0.0479 (9)	
C58	0.8165 (3)	0.2458 (3)	0.3997 (3)	0.0536 (10)	
H58	0.8822	0.2067	0.4082	0.064*	
C59	0.7771 (3)	0.3451 (3)	0.3956 (3)	0.0527 (9)	
H59	0.8101	0.3890	0.4005	0.063*	
C60	0.6761 (3)	0.3724 (3)	0.3825 (2)	0.0472 (8)	
C61	0.6104 (3)	0.4692 (3)	0.3748 (2)	0.0467 (8)	
C62	0.5093 (3)	0.4929 (3)	0.3700 (2)	0.0459 (8)	
C63	0.4407 (3)	0.5919 (3)	0.3654 (3)	0.0514 (9)	
H63	0.4565	0.6524	0.3599	0.062*	
C64	0.3489 (3)	0.5822 (3)	0.3702 (3)	0.0524 (9)	
H64	0.2881	0.6344	0.3697	0.063*	
C65	0.3610 (3)	0.4776 (3)	0.3763 (2)	0.0475 (9)	
C66	0.2824 (3)	0.4377 (3)	0.3825 (2)	0.0474 (8)	
C67	0.2946 (3)	0.3389 (3)	0.3802 (3)	0.0485 (9)	
C68	0.2149 (3)	0.2987 (3)	0.3842 (3)	0.0548 (10)	
H68	0.1459	0.3327	0.3905	0.066*	
C69	0.2568 (3)	0.2030 (3)	0.3773 (3)	0.0551 (10)	
H69	0.2224	0.1578	0.3768	0.066*	
C70	0.3625 (3)	0.1819 (3)	0.3709 (2)	0.0468 (8)	
C71	0.3930 (3)	0.0095 (3)	0.3583 (3)	0.0476 (9)	
C72	0.3945 (3)	−0.0815 (3)	0.4165 (3)	0.0530 (9)	
H72	0.4194	−0.0947	0.4644	0.064*	
C73	0.3592 (4)	−0.1533 (3)	0.4041 (3)	0.0591 (11)	
H73	0.3603	−0.2154	0.4437	0.071*	
C74	0.3231 (4)	−0.1344 (3)	0.3352 (3)	0.0615 (11)	
H74	0.2996	−0.1837	0.3273	0.074*	
C75	0.3205 (4)	−0.0441 (4)	0.2770 (3)	0.0629 (11)	
H75	0.2956	−0.0311	0.2292	0.076*	
C76	0.3552 (3)	0.0273 (3)	0.2899 (3)	0.0566 (10)	
H76	0.3527	0.0898	0.2506	0.068*	
C77	0.8533 (3)	0.0381 (3)	0.3974 (3)	0.0551 (10)	
C78	0.9336 (4)	0.0471 (6)	0.3369 (4)	0.0883 (19)	
H78	0.9244	0.0994	0.2889	0.106*	
C79	1.0274 (5)	−0.0199 (7)	0.3459 (5)	0.106 (3)	
H79	1.0821	−0.0123	0.3046	0.128*	
C80	1.0409 (5)	−0.0974 (5)	0.4149 (5)	0.093 (2)	
H80	1.1044	−0.1444	0.4210	0.112*	
C81	0.9637 (5)	−0.1051 (5)	0.4726 (5)	0.113 (3)	
H81	0.9722	−0.1580	0.5203	0.135*	
C82	0.8705 (4)	−0.0369 (5)	0.4640 (4)	0.094 (2)	
H82	0.8171	−0.0436	0.5068	0.112*	
C83	0.6503 (3)	0.5529 (3)	0.3715 (2)	0.0471 (8)	
C84	0.6136 (3)	0.6077 (3)	0.4290 (3)	0.0511 (9)	
H84	0.5627	0.5912	0.4716	0.061*	
C85	0.6509 (4)	0.6860 (3)	0.4243 (3)	0.0562 (10)	
H85	0.6255	0.7230	0.4637	0.067*	
C86	0.7252 (3)	0.7106 (4)	0.3624 (3)	0.0600 (11)	
H86	0.7501	0.7648	0.3590	0.072*	
C87	0.7629 (3)	0.6564 (4)	0.3057 (3)	0.0595 (11)	
H87	0.8137	0.6733	0.2632	0.071*	
C88	0.7266 (3)	0.5774 (3)	0.3108 (3)	0.0526 (9)	
H88	0.7541	0.5392	0.2725	0.063*	
C89	0.1794 (3)	0.5053 (3)	0.3934 (3)	0.0500 (9)	
C90	0.1151 (3)	0.5333 (3)	0.3388 (3)	0.0565 (10)	
H90	0.1363	0.5081	0.2927	0.068*	
C91	0.0208 (3)	0.5973 (4)	0.3505 (4)	0.0682 (13)	
H91	−0.0226	0.6151	0.3128	0.082*	
C92	−0.0109 (4)	0.6354 (4)	0.4164 (4)	0.0737 (15)	
H92	−0.0755	0.6804	0.4237	0.088*	
C93	0.0514 (4)	0.6079 (4)	0.4721 (4)	0.0698 (13)	
H93	0.0295	0.6334	0.5180	0.084*	
C94	0.1463 (3)	0.5429 (4)	0.4607 (3)	0.0579 (10)	
H94	0.1889	0.5239	0.4992	0.069*	

### Atomic displacement parameters (Å^2^)

**Table:**

	*U*^11^	*U*^22^	*U*^33^	*U*^12^	*U*^13^	*U*^23^
Fe1	0.0391 (3)	0.0422 (3)	0.0472 (3)	−0.0118 (2)	−0.0078 (2)	−0.0109 (2)
O1	0.0467 (14)	0.0497 (15)	0.0506 (15)	−0.0151 (12)	−0.0077 (12)	−0.0125 (12)
N1	0.0419 (17)	0.0441 (17)	0.0568 (19)	−0.0140 (14)	−0.0066 (14)	−0.0122 (14)
N2	0.0400 (16)	0.0437 (17)	0.0533 (18)	−0.0132 (13)	−0.0103 (13)	−0.0086 (14)
N3	0.0444 (17)	0.0423 (17)	0.0532 (18)	−0.0125 (14)	−0.0095 (14)	−0.0121 (14)
N4	0.0382 (16)	0.0463 (17)	0.0516 (18)	−0.0115 (13)	−0.0086 (13)	−0.0130 (14)
C1	0.042 (2)	0.048 (2)	0.063 (2)	−0.0116 (17)	−0.0065 (17)	−0.0141 (18)
C2	0.041 (2)	0.047 (2)	0.061 (2)	−0.0091 (16)	−0.0062 (17)	−0.0180 (18)
C3	0.042 (2)	0.053 (2)	0.075 (3)	−0.0097 (18)	−0.0085 (19)	−0.021 (2)
C4	0.0385 (19)	0.055 (2)	0.068 (3)	−0.0123 (17)	−0.0089 (18)	−0.020 (2)
C5	0.0386 (19)	0.049 (2)	0.054 (2)	−0.0155 (16)	−0.0059 (16)	−0.0141 (17)
C6	0.044 (2)	0.045 (2)	0.052 (2)	−0.0150 (16)	−0.0079 (16)	−0.0113 (16)
C7	0.044 (2)	0.045 (2)	0.054 (2)	−0.0142 (16)	−0.0100 (16)	−0.0096 (17)
C8	0.048 (2)	0.048 (2)	0.061 (2)	−0.0150 (17)	−0.0130 (18)	−0.0151 (18)
C9	0.048 (2)	0.046 (2)	0.065 (3)	−0.0123 (17)	−0.0116 (19)	−0.0160 (19)
C10	0.045 (2)	0.044 (2)	0.053 (2)	−0.0143 (16)	−0.0103 (16)	−0.0086 (16)
C11	0.046 (2)	0.046 (2)	0.050 (2)	−0.0136 (17)	−0.0099 (16)	−0.0100 (16)
C12	0.0430 (19)	0.044 (2)	0.053 (2)	−0.0103 (16)	−0.0119 (16)	−0.0113 (17)
C13	0.043 (2)	0.047 (2)	0.060 (2)	−0.0101 (17)	−0.0133 (17)	−0.0124 (18)
C14	0.043 (2)	0.053 (2)	0.057 (2)	−0.0130 (17)	−0.0131 (17)	−0.0151 (18)
C15	0.0389 (18)	0.047 (2)	0.051 (2)	−0.0123 (16)	−0.0090 (15)	−0.0110 (16)
C16	0.0419 (19)	0.046 (2)	0.055 (2)	−0.0155 (16)	−0.0089 (16)	−0.0098 (17)
C17	0.0424 (19)	0.046 (2)	0.052 (2)	−0.0133 (16)	−0.0082 (16)	−0.0163 (17)
C18	0.045 (2)	0.049 (2)	0.068 (3)	−0.0155 (17)	−0.0087 (18)	−0.0149 (19)
C19	0.048 (2)	0.043 (2)	0.071 (3)	−0.0125 (17)	−0.0098 (19)	−0.0156 (19)
C20	0.045 (2)	0.043 (2)	0.054 (2)	−0.0123 (16)	−0.0052 (16)	−0.0137 (16)
C21	0.040 (2)	0.049 (2)	0.078 (3)	−0.0121 (17)	−0.0098 (19)	−0.017 (2)
C22	0.065 (7)	0.040 (5)	0.069 (6)	−0.013 (4)	−0.001 (5)	−0.009 (4)
C23	0.072 (9)	0.058 (7)	0.079 (7)	−0.003 (6)	−0.024 (6)	−0.014 (6)
C24	0.054 (10)	0.035 (5)	0.078 (9)	−0.018 (5)	−0.011 (7)	−0.008 (6)
C25	0.088 (9)	0.049 (6)	0.085 (8)	−0.004 (5)	−0.033 (7)	−0.033 (5)
C26	0.083 (9)	0.057 (7)	0.086 (8)	−0.007 (5)	−0.038 (6)	−0.034 (6)
C22'	0.059 (8)	0.041 (7)	0.122 (13)	−0.020 (6)	0.021 (8)	−0.005 (7)
C23'	0.060 (9)	0.049 (8)	0.114 (18)	−0.002 (7)	−0.002 (10)	0.001 (9)
C24'	0.032 (6)	0.044 (10)	0.093 (14)	−0.013 (6)	−0.006 (9)	−0.015 (9)
C25'	0.068 (9)	0.077 (11)	0.123 (14)	−0.008 (7)	−0.022 (9)	−0.057 (10)
C26'	0.070 (9)	0.066 (10)	0.089 (10)	0.007 (7)	−0.037 (8)	−0.041 (8)
C27	0.0413 (19)	0.050 (2)	0.054 (2)	−0.0143 (16)	−0.0057 (16)	−0.0157 (17)
C28	0.056 (2)	0.055 (2)	0.065 (3)	−0.022 (2)	−0.013 (2)	−0.013 (2)
C29	0.065 (3)	0.065 (3)	0.079 (3)	−0.032 (2)	−0.003 (2)	−0.023 (2)
C30	0.048 (2)	0.073 (3)	0.090 (4)	−0.024 (2)	−0.007 (2)	−0.033 (3)
C31	0.051 (2)	0.064 (3)	0.078 (3)	−0.009 (2)	−0.020 (2)	−0.028 (2)
C32	0.045 (2)	0.054 (2)	0.064 (3)	−0.0136 (18)	−0.0110 (18)	−0.017 (2)
C33	0.0425 (19)	0.047 (2)	0.053 (2)	−0.0088 (16)	−0.0129 (16)	−0.0144 (17)
C34	0.047 (2)	0.056 (2)	0.059 (2)	−0.0151 (19)	−0.0067 (18)	−0.0150 (19)
C35	0.047 (2)	0.069 (3)	0.072 (3)	−0.009 (2)	−0.009 (2)	−0.027 (2)
C36	0.058 (3)	0.056 (3)	0.075 (3)	−0.002 (2)	−0.016 (2)	−0.026 (2)
C37	0.072 (3)	0.045 (2)	0.068 (3)	−0.011 (2)	−0.021 (2)	−0.013 (2)
C38	0.056 (2)	0.046 (2)	0.053 (2)	−0.0129 (18)	−0.0086 (18)	−0.0117 (17)
C39	0.045 (2)	0.045 (2)	0.066 (3)	−0.0146 (17)	−0.0162 (18)	−0.0076 (18)
C40	0.052 (2)	0.050 (2)	0.069 (3)	−0.0177 (19)	−0.017 (2)	−0.012 (2)
C41	0.060 (3)	0.050 (2)	0.099 (4)	−0.016 (2)	−0.031 (3)	−0.017 (2)
C42	0.055 (3)	0.048 (2)	0.109 (4)	−0.018 (2)	−0.027 (3)	−0.009 (3)
C43	0.040 (2)	0.061 (3)	0.092 (4)	−0.016 (2)	−0.011 (2)	−0.010 (3)
C44	0.043 (2)	0.054 (2)	0.072 (3)	−0.0133 (18)	−0.0118 (19)	−0.010 (2)
Fe2	0.0415 (3)	0.0400 (3)	0.0470 (3)	−0.0110 (2)	−0.0083 (2)	−0.0113 (2)
N51	0.0442 (17)	0.0418 (17)	0.0522 (18)	−0.0120 (13)	−0.0107 (14)	−0.0112 (14)
N52	0.0434 (17)	0.0402 (17)	0.0537 (18)	−0.0081 (13)	−0.0094 (14)	−0.0128 (14)
N53	0.0430 (16)	0.0405 (16)	0.0513 (17)	−0.0103 (13)	−0.0085 (13)	−0.0135 (13)
N54	0.0447 (17)	0.0420 (17)	0.0517 (18)	−0.0118 (14)	−0.0066 (14)	−0.0127 (14)
C51	0.048 (2)	0.044 (2)	0.053 (2)	−0.0151 (17)	−0.0072 (17)	−0.0126 (17)
C52	0.046 (2)	0.043 (2)	0.055 (2)	−0.0117 (16)	−0.0063 (17)	−0.0149 (17)
C53	0.054 (2)	0.044 (2)	0.069 (3)	−0.0117 (18)	−0.008 (2)	−0.0197 (19)
C54	0.048 (2)	0.044 (2)	0.079 (3)	−0.0064 (18)	−0.013 (2)	−0.020 (2)
C55	0.044 (2)	0.0409 (19)	0.055 (2)	−0.0064 (16)	−0.0122 (17)	−0.0111 (16)
C56	0.045 (2)	0.042 (2)	0.054 (2)	−0.0068 (16)	−0.0108 (17)	−0.0121 (17)
C57	0.044 (2)	0.042 (2)	0.056 (2)	−0.0096 (16)	−0.0117 (17)	−0.0114 (17)
C58	0.046 (2)	0.052 (2)	0.067 (3)	−0.0111 (18)	−0.0182 (19)	−0.017 (2)
C59	0.048 (2)	0.047 (2)	0.067 (3)	−0.0108 (17)	−0.0152 (19)	−0.0172 (19)
C60	0.048 (2)	0.0411 (19)	0.055 (2)	−0.0115 (16)	−0.0121 (17)	−0.0129 (16)
C61	0.045 (2)	0.045 (2)	0.052 (2)	−0.0121 (16)	−0.0095 (16)	−0.0150 (17)
C62	0.047 (2)	0.0420 (19)	0.053 (2)	−0.0146 (16)	−0.0119 (16)	−0.0121 (16)
C63	0.050 (2)	0.040 (2)	0.065 (3)	−0.0111 (17)	−0.0121 (19)	−0.0128 (18)
C64	0.043 (2)	0.045 (2)	0.069 (3)	−0.0081 (17)	−0.0130 (18)	−0.0157 (19)
C65	0.043 (2)	0.045 (2)	0.053 (2)	−0.0070 (16)	−0.0104 (16)	−0.0142 (17)
C66	0.044 (2)	0.046 (2)	0.052 (2)	−0.0115 (17)	−0.0095 (16)	−0.0134 (17)
C67	0.044 (2)	0.045 (2)	0.054 (2)	−0.0101 (16)	−0.0059 (16)	−0.0137 (17)
C68	0.044 (2)	0.048 (2)	0.072 (3)	−0.0116 (17)	−0.0057 (19)	−0.019 (2)
C69	0.046 (2)	0.048 (2)	0.076 (3)	−0.0191 (18)	−0.0074 (19)	−0.017 (2)
C70	0.043 (2)	0.0414 (19)	0.057 (2)	−0.0135 (16)	−0.0060 (16)	−0.0136 (16)
C71	0.046 (2)	0.0394 (19)	0.057 (2)	−0.0128 (16)	−0.0069 (17)	−0.0118 (16)
C72	0.057 (2)	0.047 (2)	0.055 (2)	−0.0181 (19)	−0.0052 (18)	−0.0125 (18)
C73	0.065 (3)	0.045 (2)	0.069 (3)	−0.022 (2)	−0.003 (2)	−0.014 (2)
C74	0.058 (3)	0.048 (2)	0.085 (3)	−0.020 (2)	−0.011 (2)	−0.020 (2)
C75	0.067 (3)	0.058 (3)	0.074 (3)	−0.019 (2)	−0.023 (2)	−0.019 (2)
C76	0.061 (3)	0.047 (2)	0.065 (3)	−0.020 (2)	−0.017 (2)	−0.0085 (19)
C77	0.049 (2)	0.048 (2)	0.071 (3)	−0.0086 (18)	−0.017 (2)	−0.019 (2)
C78	0.060 (3)	0.110 (5)	0.073 (3)	0.003 (3)	−0.010 (3)	−0.024 (3)
C79	0.052 (3)	0.142 (7)	0.102 (5)	0.010 (4)	−0.002 (3)	−0.047 (5)
C80	0.059 (3)	0.073 (4)	0.134 (6)	0.010 (3)	−0.028 (4)	−0.030 (4)
C81	0.057 (3)	0.084 (4)	0.146 (7)	−0.003 (3)	−0.027 (4)	0.028 (4)
C82	0.051 (3)	0.080 (4)	0.108 (5)	−0.005 (3)	−0.015 (3)	0.020 (3)
C83	0.045 (2)	0.044 (2)	0.054 (2)	−0.0108 (16)	−0.0116 (17)	−0.0153 (17)
C84	0.053 (2)	0.047 (2)	0.053 (2)	−0.0129 (18)	−0.0118 (18)	−0.0126 (17)
C85	0.062 (3)	0.049 (2)	0.065 (3)	−0.014 (2)	−0.018 (2)	−0.019 (2)
C86	0.054 (2)	0.050 (2)	0.084 (3)	−0.0174 (19)	−0.018 (2)	−0.020 (2)
C87	0.047 (2)	0.055 (2)	0.080 (3)	−0.0195 (19)	−0.008 (2)	−0.018 (2)
C88	0.046 (2)	0.052 (2)	0.062 (2)	−0.0142 (18)	−0.0060 (18)	−0.0202 (19)
C89	0.0382 (19)	0.044 (2)	0.068 (3)	−0.0117 (16)	−0.0070 (17)	−0.0141 (18)
C90	0.049 (2)	0.052 (2)	0.070 (3)	−0.0145 (19)	−0.013 (2)	−0.015 (2)
C91	0.045 (2)	0.059 (3)	0.096 (4)	−0.010 (2)	−0.021 (2)	−0.011 (3)
C92	0.043 (2)	0.062 (3)	0.107 (4)	−0.004 (2)	−0.009 (3)	−0.024 (3)
C93	0.053 (3)	0.061 (3)	0.095 (4)	−0.008 (2)	−0.006 (2)	−0.033 (3)
C94	0.045 (2)	0.056 (2)	0.075 (3)	−0.0114 (19)	−0.007 (2)	−0.026 (2)

### Geometric parameters (Å, º)

**Table:**

Fe1—O1	1.766 (3)	C41—H41	0.9500
Fe1—N3	2.069 (3)	C42—C43	1.374 (8)
Fe1—N2	2.078 (3)	C42—H42	0.9500
Fe1—N1	2.079 (3)	C43—C44	1.402 (6)
Fe1—N4	2.084 (3)	C43—H43	0.9500
O1—Fe2	1.757 (3)	C44—H44	0.9500
N1—C5	1.370 (5)	Fe2—N51	2.078 (3)
N1—C2	1.372 (5)	Fe2—N53	2.080 (3)
N2—C10	1.370 (5)	Fe2—N54	2.084 (3)
N2—C7	1.391 (5)	Fe2—N52	2.091 (3)
N3—C15	1.376 (5)	N51—C55	1.382 (5)
N3—C12	1.386 (5)	N51—C52	1.384 (5)
N4—C20	1.370 (5)	N52—C60	1.371 (5)
N4—C17	1.384 (5)	N52—C57	1.374 (5)
C1—C2	1.393 (6)	N53—C65	1.373 (5)
C1—C20	1.403 (6)	N53—C62	1.379 (5)
C1—C21	1.485 (6)	N54—C70	1.380 (5)
C2—C3	1.433 (6)	N54—C67	1.381 (5)
C3—C4	1.354 (6)	C51—C70	1.393 (6)
C3—H3	0.9500	C51—C52	1.407 (6)
C4—C5	1.434 (6)	C51—C71	1.494 (5)
C4—H4	0.9500	C52—C53	1.428 (6)
C5—C6	1.397 (6)	C53—C54	1.355 (6)
C6—C7	1.396 (6)	C53—H53	0.9500
C6—C27	1.497 (5)	C54—C55	1.432 (6)
C7—C8	1.429 (6)	C54—H54	0.9500
C8—C9	1.348 (6)	C55—C56	1.398 (6)
C8—H8	0.9500	C56—C57	1.399 (6)
C9—C10	1.445 (6)	C56—C77	1.494 (6)
C9—H9	0.9500	C57—C58	1.440 (6)
C10—C11	1.405 (6)	C58—C59	1.347 (6)
C11—C12	1.395 (6)	C58—H58	0.9500
C11—C33	1.485 (6)	C59—C60	1.436 (6)
C12—C13	1.437 (6)	C59—H59	0.9500
C13—C14	1.352 (6)	C60—C61	1.396 (6)
C13—H13	0.9500	C61—C62	1.402 (6)
C14—C15	1.435 (5)	C61—C83	1.491 (6)
C14—H14	0.9500	C62—C63	1.440 (6)
C15—C16	1.405 (6)	C63—C64	1.359 (6)
C16—C17	1.394 (6)	C63—H63	0.9500
C16—C39	1.496 (5)	C64—C65	1.442 (6)
C17—C18	1.437 (6)	C64—H64	0.9500
C18—C19	1.364 (6)	C65—C66	1.404 (6)
C18—H18	0.9500	C66—C67	1.401 (6)
C19—C20	1.435 (6)	C66—C89	1.491 (5)
C19—H19	0.9500	C67—C68	1.433 (6)
C21—C22'	1.337 (14)	C68—C69	1.351 (6)
C21—C26	1.357 (13)	C68—H68	0.9500
C21—C22	1.415 (11)	C69—C70	1.436 (6)
C21—C26'	1.441 (15)	C69—H69	0.9500
C22—C23	1.400 (14)	C71—C76	1.377 (6)
C22—H22	0.9500	C71—C72	1.393 (6)
C23—C24	1.389 (15)	C72—C73	1.400 (6)
C23—H23	0.9500	C72—H72	0.9500
C24—C25	1.369 (15)	C73—C74	1.368 (7)
C24—H24	0.9500	C73—H73	0.9500
C25—C26	1.387 (14)	C74—C75	1.385 (7)
C25—H25	0.9500	C74—H74	0.9500
C26—H26	0.9500	C75—C76	1.392 (6)
C22'—C23'	1.386 (16)	C75—H75	0.9500
C22'—H22'	0.9500	C76—H76	0.9500
C23'—C24'	1.394 (17)	C77—C82	1.342 (7)
C23'—H23'	0.9500	C77—C78	1.388 (8)
C24'—C25'	1.370 (16)	C78—C79	1.392 (8)
C24'—H24'	0.9500	C78—H78	0.9500
C25'—C26'	1.403 (15)	C79—C80	1.383 (10)
C25'—H25'	0.9500	C79—H79	0.9500
C26'—H26'	0.9500	C80—C81	1.329 (10)
C27—C32	1.402 (6)	C80—H80	0.9500
C27—C28	1.402 (6)	C81—C82	1.390 (8)
C28—C29	1.391 (6)	C81—H81	0.9500
C28—H28	0.9500	C82—H82	0.9500
C29—C30	1.383 (8)	C83—C88	1.394 (6)
C29—H29	0.9500	C83—C84	1.397 (6)
C30—C31	1.365 (7)	C84—C85	1.385 (6)
C30—H30	0.9500	C84—H84	0.9500
C31—C32	1.388 (6)	C85—C86	1.388 (7)
C31—H31	0.9500	C85—H85	0.9500
C32—H32	0.9500	C86—C87	1.380 (7)
C33—C34	1.389 (6)	C86—H86	0.9500
C33—C38	1.398 (6)	C87—C88	1.382 (6)
C34—C35	1.385 (6)	C87—H87	0.9500
C34—H34	0.9500	C88—H88	0.9500
C35—C36	1.378 (7)	C89—C90	1.388 (6)
C35—H35	0.9500	C89—C94	1.395 (6)
C36—C37	1.387 (7)	C90—C91	1.380 (6)
C36—H36	0.9500	C90—H90	0.9500
C37—C38	1.395 (6)	C91—C92	1.376 (8)
C37—H37	0.9500	C91—H91	0.9500
C38—H38	0.9500	C92—C93	1.383 (8)
C39—C44	1.389 (6)	C92—H92	0.9500
C39—C40	1.397 (6)	C93—C94	1.391 (6)
C40—C41	1.387 (6)	C93—H93	0.9500
C40—H40	0.9500	C94—H94	0.9500
C41—C42	1.371 (8)		
			
O1—Fe1—N3	103.31 (13)	C41—C42—C43	120.1 (5)
O1—Fe1—N2	102.11 (13)	C41—C42—H42	119.9
N3—Fe1—N2	87.38 (13)	C43—C42—H42	119.9
O1—Fe1—N1	103.37 (13)	C42—C43—C44	120.4 (5)
N3—Fe1—N1	153.32 (14)	C42—C43—H43	119.8
N2—Fe1—N1	87.14 (13)	C44—C43—H43	119.8
O1—Fe1—N4	102.12 (13)	C39—C44—C43	119.7 (5)
N3—Fe1—N4	87.27 (13)	C39—C44—H44	120.2
N2—Fe1—N4	155.77 (13)	C43—C44—H44	120.2
N1—Fe1—N4	87.10 (13)	O1—Fe2—N51	103.46 (13)
Fe2—O1—Fe1	177.71 (18)	O1—Fe2—N53	104.50 (13)
C5—N1—C2	106.6 (3)	N51—Fe2—N53	152.04 (13)
C5—N1—Fe1	126.6 (3)	O1—Fe2—N54	103.78 (13)
C2—N1—Fe1	125.4 (3)	N51—Fe2—N54	86.63 (13)
C10—N2—C7	106.2 (3)	N53—Fe2—N54	86.85 (13)
C10—N2—Fe1	123.3 (3)	O1—Fe2—N52	103.63 (13)
C7—N2—Fe1	125.1 (3)	N51—Fe2—N52	86.99 (13)
C15—N3—C12	106.1 (3)	N53—Fe2—N52	86.40 (13)
C15—N3—Fe1	127.4 (3)	N54—Fe2—N52	152.59 (13)
C12—N3—Fe1	125.6 (3)	C55—N51—C52	105.7 (3)
C20—N4—C17	106.8 (3)	C55—N51—Fe2	126.5 (3)
C20—N4—Fe1	124.3 (3)	C52—N51—Fe2	124.8 (3)
C17—N4—Fe1	125.4 (3)	C60—N52—C57	106.7 (3)
C2—C1—C20	124.0 (4)	C60—N52—Fe2	126.3 (3)
C2—C1—C21	116.8 (4)	C57—N52—Fe2	126.2 (3)
C20—C1—C21	119.2 (4)	C65—N53—C62	106.1 (3)
N1—C2—C1	126.6 (4)	C65—N53—Fe2	126.3 (3)
N1—C2—C3	109.5 (4)	C62—N53—Fe2	124.7 (3)
C1—C2—C3	123.9 (4)	C70—N54—C67	106.0 (3)
C4—C3—C2	107.2 (4)	C70—N54—Fe2	125.3 (3)
C4—C3—H3	126.4	C67—N54—Fe2	127.4 (3)
C2—C3—H3	126.4	C70—C51—C52	124.2 (4)
C3—C4—C5	107.0 (4)	C70—C51—C71	117.5 (4)
C3—C4—H4	126.5	C52—C51—C71	118.2 (4)
C5—C4—H4	126.5	N51—C52—C51	125.6 (4)
N1—C5—C6	126.0 (4)	N51—C52—C53	109.8 (4)
N1—C5—C4	109.6 (4)	C51—C52—C53	124.6 (4)
C6—C5—C4	124.3 (4)	C54—C53—C52	107.6 (4)
C7—C6—C5	124.9 (4)	C54—C53—H53	126.2
C7—C6—C27	117.9 (4)	C52—C53—H53	126.2
C5—C6—C27	117.2 (4)	C53—C54—C55	106.9 (4)
N2—C7—C6	125.0 (4)	C53—C54—H54	126.5
N2—C7—C8	109.3 (4)	C55—C54—H54	126.5
C6—C7—C8	125.6 (4)	N51—C55—C56	125.2 (4)
C9—C8—C7	107.9 (4)	N51—C55—C54	110.0 (4)
C9—C8—H8	126.1	C56—C55—C54	124.8 (4)
C7—C8—H8	126.1	C55—C56—C57	124.8 (4)
C8—C9—C10	106.9 (4)	C55—C56—C77	118.0 (4)
C8—C9—H9	126.6	C57—C56—C77	117.2 (4)
C10—C9—H9	126.6	N52—C57—C56	126.2 (4)
N2—C10—C11	125.5 (4)	N52—C57—C58	109.4 (4)
N2—C10—C9	109.8 (3)	C56—C57—C58	124.3 (4)
C11—C10—C9	124.7 (4)	C59—C58—C57	107.0 (4)
C12—C11—C10	124.4 (4)	C59—C58—H58	126.5
C12—C11—C33	117.1 (4)	C57—C58—H58	126.5
C10—C11—C33	118.4 (4)	C58—C59—C60	107.6 (4)
N3—C12—C11	125.4 (4)	C58—C59—H59	126.2
N3—C12—C13	109.4 (4)	C60—C59—H59	126.2
C11—C12—C13	125.2 (4)	N52—C60—C61	125.8 (4)
C14—C13—C12	107.3 (4)	N52—C60—C59	109.3 (3)
C14—C13—H13	126.3	C61—C60—C59	124.9 (4)
C12—C13—H13	126.3	C60—C61—C62	124.3 (4)
C13—C14—C15	107.3 (4)	C60—C61—C83	117.8 (4)
C13—C14—H14	126.3	C62—C61—C83	117.9 (4)
C15—C14—H14	126.3	N53—C62—C61	125.5 (4)
N3—C15—C16	125.3 (4)	N53—C62—C63	110.0 (3)
N3—C15—C14	109.8 (3)	C61—C62—C63	124.3 (4)
C16—C15—C14	124.9 (4)	C64—C63—C62	106.8 (4)
C17—C16—C15	125.0 (4)	C64—C63—H63	126.6
C17—C16—C39	117.3 (4)	C62—C63—H63	126.6
C15—C16—C39	117.7 (4)	C63—C64—C65	107.1 (4)
N4—C17—C16	125.3 (4)	C63—C64—H64	126.4
N4—C17—C18	109.2 (3)	C65—C64—H64	126.4
C16—C17—C18	125.5 (4)	N53—C65—C66	126.0 (4)
C19—C18—C17	107.1 (4)	N53—C65—C64	109.9 (4)
C19—C18—H18	126.4	C66—C65—C64	124.1 (4)
C17—C18—H18	126.4	C67—C66—C65	124.4 (4)
C18—C19—C20	107.2 (4)	C67—C66—C89	118.3 (4)
C18—C19—H19	126.4	C65—C66—C89	117.4 (4)
C20—C19—H19	126.4	N54—C67—C66	125.3 (4)
N4—C20—C1	126.0 (4)	N54—C67—C68	110.0 (4)
N4—C20—C19	109.7 (4)	C66—C67—C68	124.6 (4)
C1—C20—C19	124.3 (4)	C69—C68—C67	106.8 (4)
C26—C21—C22	116.4 (9)	C69—C68—H68	126.6
C22'—C21—C26'	119.0 (10)	C67—C68—H68	126.6
C22'—C21—C1	124.5 (8)	C68—C69—C70	107.8 (4)
C26—C21—C1	126.1 (7)	C68—C69—H69	126.1
C22—C21—C1	117.5 (6)	C70—C69—H69	126.1
C26'—C21—C1	116.5 (8)	N54—C70—C51	125.9 (4)
C23—C22—C21	122.0 (11)	N54—C70—C69	109.3 (3)
C23—C22—H22	119.0	C51—C70—C69	124.7 (4)
C21—C22—H22	119.0	C76—C71—C72	118.9 (4)
C24—C23—C22	119.5 (15)	C76—C71—C51	119.2 (4)
C24—C23—H23	120.3	C72—C71—C51	121.8 (4)
C22—C23—H23	120.3	C71—C72—C73	119.7 (4)
C25—C24—C23	117.4 (16)	C71—C72—H72	120.1
C25—C24—H24	121.3	C73—C72—H72	120.1
C23—C24—H24	121.3	C74—C73—C72	120.3 (4)
C24—C25—C26	122.6 (13)	C74—C73—H73	119.9
C24—C25—H25	118.7	C72—C73—H73	119.9
C26—C25—H25	118.7	C73—C74—C75	120.8 (4)
C21—C26—C25	121.6 (12)	C73—C74—H74	119.6
C21—C26—H26	119.2	C75—C74—H74	119.6
C25—C26—H26	119.2	C74—C75—C76	118.7 (5)
C21—C22'—C23'	121.7 (15)	C74—C75—H75	120.7
C21—C22'—H22'	119.1	C76—C75—H75	120.7
C23'—C22'—H22'	119.1	C71—C76—C75	121.7 (4)
C22'—C23'—C24'	118.6 (19)	C71—C76—H76	119.2
C22'—C23'—H23'	120.7	C75—C76—H76	119.2
C24'—C23'—H23'	120.7	C82—C77—C78	117.3 (5)
C25'—C24'—C23'	122 (2)	C82—C77—C56	121.6 (5)
C25'—C24'—H24'	119.0	C78—C77—C56	121.2 (5)
C23'—C24'—H24'	119.0	C77—C78—C79	120.5 (6)
C24'—C25'—C26'	117.7 (18)	C77—C78—H78	119.7
C24'—C25'—H25'	121.1	C79—C78—H78	119.7
C26'—C25'—H25'	121.1	C80—C79—C78	120.1 (6)
C25'—C26'—C21	119.9 (15)	C80—C79—H79	120.0
C25'—C26'—H26'	120.1	C78—C79—H79	120.0
C21—C26'—H26'	120.1	C81—C80—C79	118.9 (6)
C32—C27—C28	118.2 (4)	C81—C80—H80	120.6
C32—C27—C6	120.4 (4)	C79—C80—H80	120.6
C28—C27—C6	121.4 (4)	C80—C81—C82	120.8 (6)
C29—C28—C27	119.9 (4)	C80—C81—H81	119.6
C29—C28—H28	120.0	C82—C81—H81	119.6
C27—C28—H28	120.0	C77—C82—C81	122.4 (6)
C30—C29—C28	120.9 (5)	C77—C82—H82	118.8
C30—C29—H29	119.6	C81—C82—H82	118.8
C28—C29—H29	119.6	C88—C83—C84	118.5 (4)
C31—C30—C29	119.6 (4)	C88—C83—C61	120.3 (4)
C31—C30—H30	120.2	C84—C83—C61	121.3 (4)
C29—C30—H30	120.2	C85—C84—C83	120.4 (4)
C30—C31—C32	120.8 (5)	C85—C84—H84	119.8
C30—C31—H31	119.6	C83—C84—H84	119.8
C32—C31—H31	119.6	C84—C85—C86	120.1 (4)
C31—C32—C27	120.5 (4)	C84—C85—H85	119.9
C31—C32—H32	119.7	C86—C85—H85	119.9
C27—C32—H32	119.7	C87—C86—C85	120.1 (4)
C34—C33—C38	118.0 (4)	C87—C86—H86	120.0
C34—C33—C11	119.9 (4)	C85—C86—H86	120.0
C38—C33—C11	122.1 (4)	C86—C87—C88	119.8 (4)
C35—C34—C33	121.7 (4)	C86—C87—H87	120.1
C35—C34—H34	119.2	C88—C87—H87	120.1
C33—C34—H34	119.2	C87—C88—C83	121.1 (4)
C36—C35—C34	119.7 (5)	C87—C88—H88	119.5
C36—C35—H35	120.2	C83—C88—H88	119.5
C34—C35—H35	120.2	C90—C89—C94	118.5 (4)
C35—C36—C37	120.2 (4)	C90—C89—C66	122.6 (4)
C35—C36—H36	119.9	C94—C89—C66	118.9 (4)
C37—C36—H36	119.9	C91—C90—C89	120.8 (5)
C36—C37—C38	119.8 (4)	C91—C90—H90	119.6
C36—C37—H37	120.1	C89—C90—H90	119.6
C38—C37—H37	120.1	C92—C91—C90	120.5 (5)
C37—C38—C33	120.7 (4)	C92—C91—H91	119.8
C37—C38—H38	119.7	C90—C91—H91	119.8
C33—C38—H38	119.7	C91—C92—C93	119.8 (5)
C44—C39—C40	119.0 (4)	C91—C92—H92	120.1
C44—C39—C16	120.3 (4)	C93—C92—H92	120.1
C40—C39—C16	120.7 (4)	C92—C93—C94	119.9 (5)
C41—C40—C39	120.4 (5)	C92—C93—H93	120.1
C41—C40—H40	119.8	C94—C93—H93	120.1
C39—C40—H40	119.8	C93—C94—C89	120.6 (5)
C42—C41—C40	120.4 (5)	C93—C94—H94	119.7
C42—C41—H41	119.8	C89—C94—H94	119.7
C40—C41—H41	119.8		
